# The incentive role of media companies’ executive compensation system in transformation and upgrading: Evidence from listed media companies in China

**DOI:** 10.1371/journal.pone.0286729

**Published:** 2023-06-12

**Authors:** Wancheng Yang, Jinwen Xu, Yihan Zhang, Xiaodan Wei, Shaofeng Wang

**Affiliations:** School of Logistics and e-Commerce, Zhejiang Wanli University, Ningbo, China; Guangdong University of Foreign Studies, CHINA

## Abstract

Media companies in various countries are transforming and upgrading to improve their competitiveness in the digital economy. However, existing research only focuses on the issue of how media companies transform while ignoring whether internal governance mechanisms such as compensation incentives can promote corporate value during the transformation process. According to the principal-agent theory, we examined the incentive effects of the executive compensation system in terms of monetary compensation, equity compensation, and perks in a sample of Chinese media companies in the process of transformation and upgrading. The results have revealed that monetary compensation does not have a significant incentive effect, and equity compensation and perks have an incentive effect when they are in the suitable range. Based on the results, we proposed policy recommendations from three aspects: monetary compensation, equity compensation, and perks. This study complements the research content on the executive compensation system in media enterprises’ transformation and upgrading. It can provide a reference for setting the administrative compensation system for media companies in China and other emerging economies.

## 1. Introduction

As an indispensable part of the social system, the media industry contributes to the rapid development of other related industries [1]. However, from a global perspective, the media industry has undergone tremendous changes in both market structure and competitive environment due to the impact of Internet technology [2,3]. In various countries, media industries are actively implementing transformations and upgrades to enhance their competitive advantage in the digital age. Media enterprises are important micro subjects in implementing industrial transformation and upgrading, and their executives play a leading role in the transformation and upgrading activities. According to principal-agent theory, executives will choose non-corporate value-maximizing behaviors that benefit their positions and interests [4], resulting in a decline in corporate performance. Therefore, establishing an effective governance system to alleviate the agency problem and promote improving corporate performance has become an essential issue of corporate governance in transforming and upgrading media companies.

According to the interest convergence hypothesis [5], in the decision-making of weighing the short-term interests and long-term development of shareholders and managers, setting up an effective compensation system can make the interests of executives converge with shareholders [6], thereby alleviating the agency problem. However, the debate on executive compensation has been ongoing for a long time. Some scholars believe that excessive executive compensation wastes corporate resources and increases the income gap between executives and ordinary employees, which could be more conducive to corporate development [7]. Furthermore, the excessive compensation will give executives more power, enabling them to implement non-value-maximizing decisions [8,9]. Accordingly, setting up an effective compensation system to alleviate the agency problem between shareholders and executives in the long and short term, and to increase the core competitiveness of the enterprise, has become the focus of work for the shareholders of media companies.

A typical compensation system mainly includes monetary and equity compensation [10–12]. The executives of media companies’ income mostly come from short-term monetary compensation, and the amount of compensation depends on the level of short-term operating performance. However, the transformation and upgrading of media companies are characterized by long cycles, high risks, and lagging returns [13,14]. It takes work to achieve rapid performance growth in the short term. At this time, the monetary compensation system will directly affect the enthusiasm of executives [15]. At the same time, executives must contiguously change crucial areas, such as media content, in transforming and upgrading media companies [16]. Adequate equity compensation can make executives and shareholders a long-term community of interests as the fundamental guarantee of corporate value in the long-term transformation and upgrading process. In addition, in China and other emerging markets, due to the imperfect executive compensation system, the on-the-job consumption and benefits enjoyed by executives through positions are usually considered invisible compensation that can make up for the shortcomings of the existing compensation system [17]. According to the management power theory, more than monetary and equity compensation given to executives is needed to solve the agency problem. However, it is a concrete manifestation of the agency problem [9], and the role of invisible compensation, such as job benefits, is also mixed [18–20]. As the primary system in corporate governance, can media companies’ executive compensation systems, such as monetary compensation, equity compensation, and invisible compensation, play an active role in the process of transformation and upgrading? The answers to the above questions have essential theoretical and practical significance for media companies to establish an effective salary system during the transformation and upgrading period.

The original purpose of establishing a compensation system for executives is to alleviate the principal-agent problem [5]. It is necessary to select the economic behaviors of media companies with more prominent principal-agent conflicts in the transformation process and upgrade the sample to make the research results more accurate. Moeller (2002) found that principal-agent conflicts would be intensified during the M&A process [21]. Based on this, in the context of M&A, it can be more accurately judged whether the salary system has an incentive effect. In addition, the media industry has been transforming and upgrading since 2004 in China. With the innovation and reform of the media system [22,23], the media industry is now showing an incredible pace of development following the 13th 5-year plan of the Chinese government to single it out as the primary industry of the national economy [24], it has now demonstrated a strong development vitality [25].

Moreover, most Chinese media companies will use M&A to gain competitive advantages against new media quickly and achieve transformation and upgrading. Accordingly, Chinese media companies in the process of transformation and upgrading provide a good sample for studying the incentive effect of the compensation system. This study puts the research on the effectiveness of the compensation system in the context of M&A. Referring to studies such as Zhang et al. (2022) [11], and the particular situation in which perks are used as invisible income in the Chinese market [20,26], we used monetary compensation, equity compensation, and perks as the essential components of the executive compensation system and explored its incentive effect.

The contributions are as follows. Firstly, more works of literature explore the issues in the process of transformation and upgrading of the media industry from a macro perspective, such as the current situation and future direction [27], employee involvement [28], and the key areas of transformation [13]. However, media companies will be affected by the subjective choices of executives in the process of transformation and upgrading. However, few works of literature discuss whether the executive compensation system can effectively alleviate or avoid the short-sighted behavior of executives. Based on the background of the Chinese media industry transition, this study explored whether the executive compensation system has an incentive effect and provided a helpful reference for optimizing corporate governance. Second, most literature studies the incentive effect of executive compensation from two aspects of monetary and equity compensation. However, perks in China and other transitional economies are often regarded as an invisible income when the compensation system could be better. However, there are few literature studies on its specific role in transforming and upgrading media companies. We analyzed whether perks have an incentive effect and help to establish a suitable supplementary system in the case of an imperfect executive compensation system in media companies.

The rest of this study is organized as follows: Section 2 reviews the relevant literature and proposes research hypotheses. Section 3 describes the sample data source, research models, and variables. Section 4 introduces the results of the empirical analysis. Section 5 discusses the observed effects. Section 6 presents the results of additional analysis and discusses the results. Section 7 summarized this study, recommendations, limitations and prospects.

## 2. Literature review and research hypotheses

### 2.1 The incentive effect of monetary compensation

According to the principal-agent theory, the incentive effect produced by the monetary compensation system will bring about the improvement of enterprise performance [29]. Referring to Feng et al. (2015) [30] and Lovett et al. (2022) [31], we proposed research hypotheses on whether executive salaries have an incentive effect by analyzing the impact of monetary compensation on M&A performance. M&A performance is the introductory way to measure the level of M&A transactions, which is affected by M&A target, M&A price, and M&A integration [32,33]. According to the optimal compensation contract theory, whether executives can obtain relatively fair monetary compensation will directly affect their enthusiasm and responsibility for work [34,35]. Before M&A, executives with higher monetary compensation will be strongly motivated to screen M&A targets to ensure the rationality and efficiency of M&A, and M&A performance will be more acceptable [36,37]. The appropriate M&A price requires the principal and merging executives to collect sufficient information to judge the value of the subject matter [38]. However, companies with insufficient executive monetary compensation are likelier to have higher M&A premiums [39,40]. In addition, the monetary compensation of executives is highly correlated with their size [41]. When the monetary compensation of senior executives is low, the principal-agent conflict will increase sharply. Executives can expand the company size through mergers and acquisitions, highlight the value of their human capital, and improve their salaries [42]. In this situation, performance becomes a secondary consideration for executives [43]. In the long term, the incremental enterprise value conceals many other costs in M&A, such as acquisition premiums, expected returns from debt and equity investors, and additional resource requirements to achieve synergies and value growth [44]. Shareholders must provide more monetary compensation to executives to encourage them to go beyond their typical job responsibilities and actively and significantly improve management performance [45]. Based on the above analyses, the following research hypothesis is proposed.

Hypothesis 1. During the transformation and upgrading period, the monetary compensation of media companies has an incentive effect.

### 2.2 The incentive effect of equity compensation

Like monetary compensation, according to the principal-agent theory, the final effect of the incentive effect produced by the equity compensation system is to improve corporate performance [5]. Referring to the relevant research of Lahlou & Navatte (2017) [46] and Sharma et al. (2023) [47], we analyze the impact of equity compensation on M&A performance and propose a research hypothesis on whether the system has an incentive effect. As a short-term incentive, monetary compensation cannot effectively link the long-term interests of executives and shareholders. Equity compensation encourages executives and shareholders to become a long-term interest community [48]. However, in actual research, there are two completely different conclusions about the impact of equity incentives.

On the one hand, equity incentives can reduce executives’ short-sighted and self-interested behaviors [5,49]. Companies with higher equity compensation have lower premiums during mergers and acquisitions, and the growth of acquisition targets is better [50]. Before or after mergers and acquisitions, companies will face risks such as new competitors and merger target integration. An effective equity compensation system can improve companies’ risk tolerance [51]. In addition, Chinese media companies generally have the phenomenon of "one share dominance" [52]. Equity compensation makes executives become a member of small and medium shareholders, and they will have enormous enthusiasm to resist Misguided by major shareholders [53], preventing “tunneling behavior” by major shareholders [54]. On the other hand, according to the management defense hypothesis, when executives’ shareholding exceeds a certain level, executives’ internal and external supervision and restraint will decrease [55,56]. Executives can use defacto control to seek non-monetized income to improve their returns [57]. Executing implement non-value-maximizing behaviors will not jeopardize their status and compensation [58], but enterprise value may suffer. Based on the above analyses, the following research hypothesis is proposed.

Hypothesis 2: There is an inverted U-shaped relationship between executive equity compensation and M&A performance. That is, during the transformation and upgrading of media companies, equity compensation within a specific range has an incentive effect. In contrast, excessive equity compensation will bring about a decline in the incentive effect.

### 2.3 The incentive effect of perks

The specific manifestation of Perks’ incentive effect is that it will cause changes in corporate performance [59]. Referring to the relevant research of Shi et al. (2022) [20], we analyze the relationship between Perks and M&A performance to propose the research hypothesis of whether perks have an incentive effect. Perks, also known as on-the-job consumption, refer to monetary and other consumption generated by executives in exercising power and performing duties. It is a product of regular operation and an imperfect contract product [18]. First, perks can reveal executives’ social status, identity, and prestige. It helps executives gain recognition and respect from people inside and outside the company and enables executives to obtain spiritual satisfaction and play a solid non-material incentive role [60]. Second, perks can supplement the insufficient incentives caused by inherent defects such as salary regulation [61–63]. Third, executives’ personal connections and social relations play an essential role in promoting the development of companies in China [64]. Perks are vital for maintaining executives’ social relations becomes particularly important [65]. However, perks also have the property of private benefits [66], and excessive perks may damage enterprise value [67]. Through perks, executives can obtain or enjoy full benefits at a small cost [68]. It makes executives motivated and capable of expanding the company’s scale through excessive investment, increasing perks, and maximizing private interests [69]. Accordingly, the Chinese government has kept the perks of state-owned enterprises in relevant policies, indicating that reasonable perks are authorized in China. The reason for the two contradictory economic consequences of perks is whether perks are excessive [70]. Based on the above analyses, the following research hypothesis is proposed.

Hypothesis 3: There is an inverted U-shaped relationship between executive perks and M&A performance. It suggests perks within a specific range have an incentive effect, and the incentive effect of excessive perks will decrease.

## 3. Methodology

### 3.1 Data collection and processing

The paper takes the M&A events of listed media companies from January 2008 to December 2017 as a research candidate sample. This time range considers two factors: First, the research model involves the accounting indicators of the year before the M&A announcement, and China began implementing new accounting standards in 2007. It ensures the consistency of accounting indicators; the starting point was set to 2008. Second, the long-term performance of mergers and acquisitions needs to examine in the market response two years after the announcement date of the consolidation. Since China’s significant public health emergency in 2020 will considerably impact the market, the inspection period is set to December 2019, and the latest data was collected in December 2017.

We used the CSMAR Database to count the M&A events of 118 media companies listed in China’s Shenzhen and Shanghai A-shares before 2017. The following cleaning rules are established based on data availability: (1) Exclude the specific announcement time that cannot be obtained.; (2) Exclude M&A events in which the acquirer is not a listed media company; (3) Due to the particularity of cross-border mergers and acquisitions, M&A events in which the target company is registered outside the Chinese Mainland are excluded. At the same time, the cleaning rules established to eliminate the influence of noise data are as follows: (1) Exclude M&A events with transaction capital less than 5 million, or equity transaction less than 5%, or that cannot change the controlling position of the acquiring company; (2) Exclude unfinished M&A events. Finally, 147 valid M&A events were obtained as research samples. In addition, the financial data used in this study are mainly from the annual financial statements of listed media companies.

### 3.2 Research model

#### 3.2.1 Measurement of M&A performance

M&A performance possesses short-term performance and long-term performance. We used the cumulative abnormal return (Car) based on the market model to measure short-term performance. We take the 180 to 30 trading days before the M&A announcement as the estimation window and calculate the AR of 10 trading days before and after the announcement date. In Table 1, the average AR from the announcement date to the 10th trading day is significantly different from 0, so we selected 10 trading days after the M&A announcement to calculate the CAR.

**Table 1:** Average CAR for 10 trading days before and after the M&A announcement day.

Trading day	Mean	Trading day	Mean	Trading day	Mean
- 10	-0.0004(-0.15)	-3	0.0005(0.07)	4	0.0425***(2.45)
-9	0.001(0.26)	-2	0.0055(0.73)	5	0.0422**(2.27)
-8	0.001(0.22)	-1	0.0017(0.22)	6	0.0431**(2.21)
-7	0.0005(0.1)	0	0.0201**(2.11)	7	0.0443**(2.17)
-6	-0.0013(-0.22)	1	0.0306***(2.56)	8	0.0435**(2.05)
-5	0.0003(0.06)	2	0.0366***(2.61)	9	0.0434**(1.99)
-4	0.0027(0.42)	3	0.0396***(2.43)	10	0.0377**(1.7)

Note:

*, **, *** indicate significant at 10%, 5%, 1% levels, respectively.

We use Buy-Hold abnormal return (BHAR) within 24 months to measure long-term performance. ${BHAR}_{24}=\prod_{t=1}^{24}\left(1+R_{t}\right)-\prod_{t=1}^{24}\left(1+R_{pt}\right)$, where *R*_*pt*_ is the abnormal return of the sample company in month t.

#### 3.2.2 Measurement of executive compensation

It can be seen from the relevant literature that basic executive compensation mainly includes monetary compensation and equity compensation [10–12]. At the same time, in China, the perks are regarded as an invisible income. It is often used to make up for the shortcomings of the current executive compensation system. So, we used the three indicators of monetary compensation, equity compensation, and perks to measure the executive compensation system. Among them, monetary compensation is calculated by the natural logarithm of the total salary of the top three executives. Management’s shareholding ratio measures equity compensation. As the listed media companies do not disclose specific information about perks, we referred to the method of Adithipyangkul et al. (2011) [26], which measured perks by the sum of office expenses, travel expenses, business entertainment expenses, communication expenses, training expenses, and board expenses. The ratio of perks to operating income is used as a substitute variable for perks.

#### 3.2.3 Regression model

This study established the following regression models and tested Hypotheses 1, 2, and 3 to analyze the relationship between the executive compensation system and the M&A performance of listed media companies

$${MA\_P}_{t}=\alpha_{0}+\alpha_{1}{Salary}_{t}+\alpha_{2}{Size}_{t-1}+\alpha_{3}{Tobin\prime Q}_{t-1}+\alpha_{4}{Liquidity}_{t-1}+\alpha_{5}{ROA}_{t-1}+\alpha_{6}{Lev}_{t-1}+\alpha_{7}{Top1}_{t}+\alpha_{8}{Indep}_{t}+\alpha_{9}{Gov}_{t}+\alpha_{10}{Duality}_{t}+\alpha_{11}{Property}_{t}+\alpha_{12}{Exp}_{t}+\alpha_{13}{Relevance}_{t}+\alpha_{14}{Paytype}_{t}+\sum \alpha_{i}{Year}_{i}+\varepsilon$$ (1)

$${MA\_P}_{t}=\alpha_{0}+\alpha_{1}{Equity}_{t}+\alpha_{2}{Equity}_{t}^{2}+\alpha_{3}{Size}_{t-1}+\alpha_{4}{Tobin\prime Q}_{t-1}+\alpha_{5}{Liquidity}_{t-1}+\alpha_{6}{ROA}_{t-1}+\alpha_{7}{Lev}_{t-1}+\alpha_{8}{Top1}_{t}+\alpha_{9}{Indep}_{t}+\alpha_{10}{Gov}_{t}+\alpha_{11}{Duality}_{t}+\alpha_{12}{Property}_{t}+\alpha_{13}{Exp}_{t}+\alpha_{14}{Relevance}_{t}+\alpha_{15}{Paytype}_{t}+\sum \alpha_{i}{Year}_{i}+\varepsilon$$ (2)

$${MA\_P}_{t}=\alpha_{0}+\alpha_{1}{Perks}_{t}+\alpha_{2}{Perks}_{t}^{2}+\alpha_{3}{Size}_{t-1}+\alpha_{4}{Tobin\prime Q}_{t-1}+\alpha_{5}{Liquidity}_{t-1}+\alpha_{6}{ROA}_{t-1}+\alpha_{7}{Lev}_{t-1}+\alpha_{8}{Top1}_{t}+\alpha_{9}{Indep}_{t}+\alpha_{10}{Gov}_{t}+\alpha_{11}{Duality}_{t}+\alpha_{12}{Property}_{t}+\alpha_{13}{Exp}_{t}+\alpha_{14}{Relevance}_{t}+\alpha_{15}{Paytype}_{t}+\sum \alpha_{i}{Year}_{i}+\varepsilon$$ (3)

The MA_P represents M&A performance, the Salary represents executive monetary compensation, the Equity represents executive equity compensation, and the Perks represent executives’ work allowances. The regression model also controls for financial characteristics, governance structures, and M&A transactions (Table 2). Among them, the control variables of economic aspects are lagged by one period to reduce the impact of endogenous.

**Table 2:** Control variable information.

Categories	Variable name	symbol	Variable description
Financial characteristics control variable	Company Size	Size	Natural logarithm of total assets
	Investment Opportunities	TobinQ	Company’s Tobins ’ Q
	cash flow	Liquidity	(cash flow—the sum of monetary funds and trading financial assets)/total assets
	Profitability	ROA	Net Profit/Total Assets
	corporate leverage	Lev	Assets and liabilities
Governance structure control variable	Ownership concentration	TOP 1	The shareholding ratio of the largest shareholder
	The proportion of independent directors	Indep	Number of Independent Directors/Number of Board Members
	government control	Gov	Use state-owned share ratio instead
	two jobs	Duality	Whether the chairman and the general manager are concurrently held at the same time, if it is 1, otherwise it is 0
	property rights	Property	State-owned property is 1, and private property is 0
Transaction Features control variable	Relative M&A Size	MaSize	Amount of M&A transactions/Total assets at the end of the year before M&A
	Whether related mergers and acquisitions	Related	Dummy variable, 1 for related mergers and 0 for unrelated mergers
	Transaction payment method	Pay	Dummy variables, cash payout, is 1, the stock payout is 2, the mixed payout is 3

## 4. Results

### 4.1 Descriptive statistical

We used the Winsorize method to reduce the continuous index data by 1%, and the descriptive statistics of each variable are obtained, as shown in Table 3. The average value of short-term performance (Car) and long-term performance (Bhar) are 0.036 and -0.451. Respectively, the short-term performance gap is small, while the long-term is significant. The average salary value is 5.217, the minimum value is 3.798, and the maximum is 6.409. The average weight of equity incentive is 12.917, the minimum value is 0, and the maximum value is 57.311. The average value of perks is 2.532, the minimum value is 0.007, and the maximum value is 10.551. There is a particular gap in the Salary and Equity of the sample companies, and perks generally exist in each company.

**Table 3:** Descriptive statistics.

Label	Mean	Minimum	Maximum value	Standard deviation
Car	0.036	-0.383	0.406	0.144
Bhar	-0.451	-4.387	3.089	0.871
Salary	5.217	3.798	6.409	0.561
Perks	2.532	0.007	1 0.551	2.034
Equity	12.917	0.000	57.311	16.797
Size	21.651	18.219	23.553	0.877
TobinQ	2.467	0.255	10.032	1.571
Liquidity	0.067	-0.312	0.291	0.088
ROA	6.949	-6.738	31.141	4.648
Lev	28.942	1.778	90.475	17.097
TOP 1	34.839	7.089	75.779	16.516
Indep	37.4	25	6 0	0.053
Gov	10.776	0	77.286	21.916
MaSize	42.831	0.023	1345.237	123.611
Symbol	Variable classification		Quantity	Proportion
Duality	1		92	62%
	0		57	38%
Property	1		57	38%
	0		92	62%
Related	1		57	38%
	0		92	62%
Pay	1		94	63%
	2		7	5%
	3		48	32%

### 4.2 Regression results

The regression results of the models are presented in Table 4, and the consequences of further tests of the non-linear relationship using Stata’s U-test tool are displayed in Table 5. Due to space reasons, the regression coefficients corresponding to the control variables were not listed.

**Table 4:** Executive compensation system and M&A performance regression results.

Label	M&A Short-Term Performance (Car)			M&A Long-Term Performance (Bhar)		
	Model (1)	Model (2)	Model (3)	Model (1)	Model (2)	Model (3)
Salary	-0.0863***(-5.82)			0.0186(0.49)		
Equity		0.0090***(5.52)			0.0100**(2.41)	
Equity^2^		-0.0003***(-8.34)			-0.0002**(-2.08)	
Perks			0.0967***(6.27)			0.1028***(3.03)
Perks^2^			-0.0095***(-5.51)			-0.0092**(-2.25)
Control	YES	YES	YES	YES	YES	YES
Year	YES	YES	YES	YES	YES	YES
Adj. R^2^	0.341	0.457	0.378	0.265	0.268	0.271

Note:

*, **, *** indicate significant at 10%, 5%, 1% levels, respectively.

**Table 5:** Executive compensation system and M&A performance U-test results.

Detection value	Car		Bhar	
	Perks	Equity	Perks	Equity
left slope	0.097***(6.27)	0.009***(5.52)	0.103***(3.03)	0.01***(2.41)
right slope	-0.104***(-4.69)	-0.029***(-9.43)	-0.091**(-1.65)	-0.012**(-1.77)
inflection point	5.1	14.88	5.59	28.28
minimum	0.007 _	0	0.007	0
maximum value	10.5 51	57.311	10.5 51	57.311

Note:

*, **, *** indicate significant at 10%, 5%, 1% levels, respectively.

The regression coefficient of Salary in the model (1) is significantly negative, indicating a negative correlation between Salary and M&A short-term performance. The regression coefficient of Equity in the model (2) is significantly positive, and the coefficient of equity2 is incredibly negative. The u-test illustrated that the slope of the left end is significantly positive, the right end is significantly negative, and the inflection point is 14.88, within the range of the sample’s equity. It demonstrates a significant "inverted U-shaped" relationship between equity and short-term performance. The regression coefficient of perks in the model (3) is significantly positive, while the coefficient of perks2 is significantly negative. The u-test illustrated slope of the left end is 0.097, and the slope of the right end is -0.104. These slopes are quite different from 0. The inflection point is 5.1, within the range of the sample’s perks, indicating a significant "inverted U-shaped" relationship between perks and short-term performance. The regression coefficient of salary in the model (4) is insignificant. Similarly, model (5) and the U-test results show a significant "inverted U-shaped" relationship between equity and long-term performance. According to model (6) and the U-test results, an effective "inverted U-shaped" relationship exists between perks and long-term performance.

### 4.3 Endogeneity mitigation

In the regression model, the control variables of financial characteristics are lagged by one period to reduce the impact of endogenous. In addition, we also used instrumental variable methods to reduce endogeneity. In the case of difficulty obtaining external instrumental variables, Lewbel (2012) provides a way of building instrumental variables based on Heteroscedasticity [71]. Accordingly, we used the method proposed by Lewbel (2012) in the model to construct instrumental variables and display the 2SLS regression results in Table 6. In each model, the coefficients and prominences of Salary, Equity, Equity^2^, Perks, and Perks^2^ are consistent with the results of the basic regression in Table 4.

**Table 6:** Instrumental variable estimation.

Variable symbol	M&A Short-Term Performance (Car)			M&A Long-Term Performance (Bhar)		
	Model (1)	Model (2)	Model (3)	Model (1)	Model (2)	Model (3)
Salary	-0.039***(-4.13)			0.004(0.13)		
Equity		0.003**(2.47)			0.007*(1.89)	
Equity ^2^		-0.0001***(-4.46)			-0.0001*(-1.72)	
Perks			0.059***(4.32)			0.127***(3.68)
Perks^2^			-0.01***(-5.30)			-0.018***(-3.95)
Control	YES	YES	YES	YES	YES	YES
Year	YES	YES	YES	YES	YES	YES
R ^2^	0.261	0.272	0.275	0.268	0.269	0.270

Note:

*, **, *** indicate significant at 10%, 5%, 1% levels, respectively.

### 4.4 Robustness detection

We used the method of proxy variables to test the robustness to ensure the reliability of the results. Specifically, Specifically, we used BHAR to measure short-term performance and CAR to measure long-term performance. The results of the robustness test are shown in Table 7. The significance of the regression coefficients of each explanatory variable is consistent with the results in Table 4, indicating that the conclusions of this study are robust and reliable.

**Table 7:** Incentive system and M&A performance robustness test results.

Variable symbol	M&A Short-Term Performance (Car)			M&A Long-Term Performance (Bhar)		
	Model (1)	Model (2)	Model (3)	Model (1)	Model (2)	Model (3)
Salary	0.1408***(-6.41)			0.0297(1.30)		
Equity		0.0121***(5.02)			0.0738***(3.62)	
Equity ^2^		-0.0004***(-7.25)			-0.0061**(-2.47)	
Perks			0.1700***(8.13)			0.0123***(4.32)
Perks^2^			-0.0177***(-7.63)			-0.0002***(-3.13)
Control	YES	YES	YES	YES	YES	YES
Year	YES	YES	YES	YES	YES	YES
Adj. R ^2^	0.313	0.387	0.353	0.338	0.273	0.347

Note:

*, **, *** indicate significant at 10%, 5%, 1% levels, respectively.

## 5. Discussion

The regression results reveal that executive monetary compensation does not positively affect M&A performance, so hypothesis 1 is invalid. Hillier et al. (2020) have shown that the monetary compensation system, as the most direct incentive method, can enhance executives’ work enthusiasm and promote executives’ efforts to improve corporate value [72]. However, the results of this study show that the monetary compensation system of listed media companies in China is ineffective during corporate transformation and upgrading. According to the principal-agent theory, executives have the motivation and ability to drive mergers and acquisitions for personal gain [42]. This study used the sum of the top 3 executive salaries to measure the monetary compensation system. Executives’ salaries are proportional to their power [73]. So executives with higher salaries tend to have more energy and opportunities to perform actions such as M&A to satisfy personal benefits [74] during corporate transformation and upgrading. In the transformation and upgrading of media companies, there are concerns that executives with higher salaries are making M&A to satisfy their benefits. In the long term, the executive monetary compensation fails to promote performance and exhibits a state of failure.

**Fig 1:**
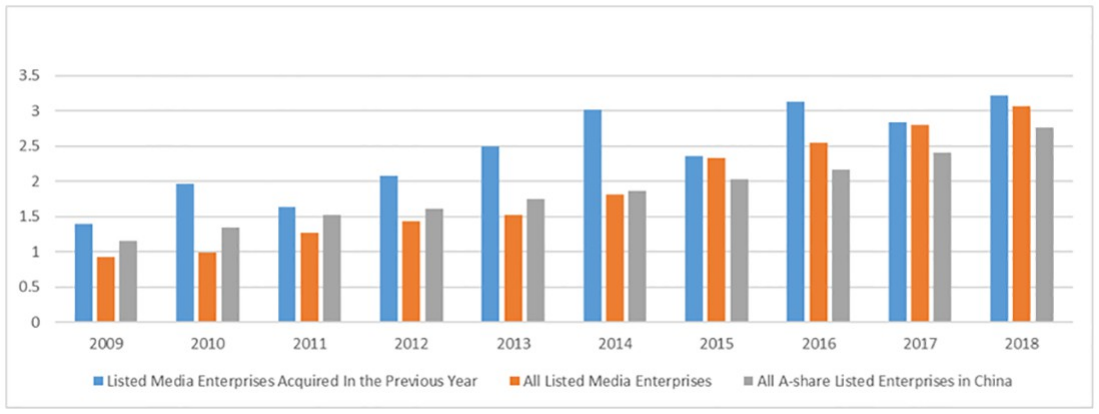
The average value of the sum of the top three executives’ remuneration (Million RMB).

We conducted statistics on the average total salary of the top three executives of listed media companies, all listed companies (excluding financial listed companies), and listed media companies acquired after one year (Fig 1). It can be seen that one year after the implementation of the M&A, the average value of the total salaries of the top three executives of the sample companies is higher than the corresponding value of listed media companies in the same period and even higher than the value of listed companies.

In China, executive salaries tend to be related to the size and management difficulty of the company [75] and have little correlation with corporate behavior, such as M&A during transformation and upgrading [21]. Executions of Chinese listed media companies are motivated to expand their companies size during the transition and upgrade to highlight the value of their human capital and increase their rights and salaries. At this point, the transformation and upgrading of the company provide a reasonable opportunity for executives to increase their salaries while improving corporate performance is no longer critical to executives. The monetary compensation system implemented for executives during the transformation and upgrading of Chinese-listed media companies must alleviate the principal-agent problem. However, it is more likely to cause agency conflicts.

Equity compensation has a significant “inverse U-shaped" effect on M&A performance, and Hypothesis 2 passed the test. Equity compensation can effectively promote short-term performance when executive shareholding is no more than 14.88%, and it can significantly extend long-term performance when executive shareholding is not higher than 28.28%. The original intention of setting up the equity compensation is to encourage executives and shareholders to become a long-term community of interest [5] and reduce agency costs. According to the results of this study, the incentive effect of equity compensation began to decline when the management held too many shares. The result confirms the conclusions of Jelinek & Stuerke (2009) and Kuo et al. (2014) [76,77]. During the transformation and upgrading of media companies, equity compensation has played an active role in optimizing risk appetite and achieving convergence of benefits. However, when equity incentives are too strong, executives are more likely to engage in speculative behavior [78]. According to the management defence hypothesis, as executives hold more shares, executives will have a powerful position in the firm, and the constraints on them will be reduced [55,58], which allows them to drive non-value maximizing decisions during the transition and to upgrade due to the pursuit of their benefits. The finding supports both optimal contracting and managerial power theories, with the former dominating at a low level of equity compensation and the latter dominating at a high level of equity compensation.

There is a significant "inverse U-shaped" relationship between perks and M&A performance, and Hypothesis 3 passed the test. According to the results, when the proportion of perks in the total revenue does not exceed 5.1%, it can effectively promote short-term performance. Moreover, it can effectively promote long-term performance when the proportion does not exceed 5.59%. Adithipyangkul et al. (2011) and Cheng et al. (2018) found that the incentive effect of perks is relatively simple [26,28]. The results of this study reflect the non-linear characteristics of the incentive effect of perks. When the perks exceed a certain level, its incentive effect will decrease. This result confirms Chen (2017)’s view that perks have a complex incentive effect [79]. The perks widely exist in companies, especially in the "relationship society", where executives use perks to maintain their relationships with the government, banks, and business partners to allow the company to get more government and social resources and to obtain more tax and fees reductions, bank loans, and business orders [80–82]. Therefore, using perks as an implicit complement to the executive compensation system is reasonable. When executives have sufficient on-the-job consumption, their desire to gain invisible income through excessive investment in corporate transformation and upgrading will be weakened, which can promote the company’s long-term development to a certain extent [70]. However, in addition to regular spending on business activities through perks, executives can also use it for personal expenditures. Due to information asymmetry, executives in a dominant information position satisfy their consumption and enjoyment through perks. So, Excessive perks are a concrete manifestation of the rent-seeking behavior of executives, which means that executives consume corporate resources to increase their interests under egoistic motives. At this time, executives use limited resources to engage in excessive on-the-job consumption behaviors such as self-enjoyment and flattering superior officials in the process of corporate transformation and upgrading, which will inevitably lead to a waste of resources and cause a decline in corporate performance.

## 6. Additional analysis

In transforming and upgrading media enterprises, M&A often brings significant risks [83] (Fisman et al., 2022). Therefore, in addition to salary, equity, and implicit income, specific risk incentive plans are often considered in compensation plans for senior executives, including stock options that encourage managerial risk-taking and pensions that discourage risk-taking [84]. After analyzing the stock options data in the CSMAR database, we found that stock options were not implemented in the companies corresponding to the sample events. Therefore, we used model (4) to analyze the role of pensions in the executive compensation system.

$${MA\_P}_{t}=\alpha_{0}+a_{1}{Pensions}_{t}+a_{2}{Size}_{t-1}+\alpha_{3}{Tobin\prime Q}_{t-1}+\alpha_{4}{Liquidity}_{t-1}+\alpha_{5}{ROA}_{t-1}+\alpha_{6}{Lev}_{t-1}+\alpha_{7}{Top1}_{t}+\alpha_{8}{Indep}_{t}+\alpha_{9}{Gov}_{t}+\alpha_{10}{Duality}_{t}+\alpha_{11}{Property}_{t}+\alpha_{12}{Exp}_{t}+\alpha_{13}{Relevance}_{t}+\alpha_{14}{Paytype}_{t}+\sum \alpha_{i}{Year}_{i}+\varepsilon$$ (4)

In model (4), pension is the executives’ pension during the acquisition period. Referring to the methods of Shan (2017) [85] and Cui (2018) [86], we use the sum of the ending balance of social insurance premiums, housing provident fund, and annuity as alternative indicators for pension and algorithmize this indicator in regression analysis.

Based on the results of the basic regression and robustness test in Table 8, we can see that Pensions have no significant effect on short-term performance but a significant adverse effect on long-term performance. The robustness test results are consistent with those of basic regression, which indicates that the result is robust. The results of this study are significantly different from those of Bhabra et al. (2022) [87], mainly because the sample of this study is Chinese media enterprises. In transforming and upgrading Chinese enterprises, executives need to have a certain sense of risk-taking [88]. The transformation and upgrading process involves risks such as market unpredictability and transaction uncertainty, and risk-taking executives will actively look for opportunities that could lead to performance improvement [89]. However, excessive pensions will inhibit the risk-taking spirit of media executives and implement conservative transformation and upgrade strategies [90]. Under this strategy, the selected acquisition target will not bring more heterogeneous cash flow in the long run, which will not be conducive to performance improvement.

**Table 8:** Pensions and M&A performance regression results.

Variable symbol	Model (4) Regression results		Model (4) Robustness detection	
	Short-Term	Long-Term	Short-Term	Long-Term
Pensions	-0.0018(-0.49)	-0.0448***(-5.97)	0.0012(0.24)	-0.0195***(-4.10)
Size	-0.0162(-1.49)	-0.0001(-0.01)	-0.0258*(-1.82)	-0.0567***(-3.79)
TobinQ	-0.0093(-1.54)	-0.0450***(-3.86)	-0.0092(-1.13)	-0.0448***(-6.06)
Liquidity	0.6196***(7.81)	0.7965***(4.45)	0.8615***(8.44)	0.4663***(4.11)
ROA	-0.0093***(-6.11)	-0.0098***(-2.83)	-0.0117***(-6.58)	-0.0058***(-2.66)
Lev	-0.0017***(-3.96)	-0.0004(-0.37)	-0.0017***(-3.03)	-0.0001(-0.20)
TOP1	0.0007(1.25)	0.0070***(6.82)	-0.0005(-0.76)	0.0044***(6.83)
Indep	-0.0074***(-5.50)	0.0079***(2.89)	-0.0096***(-5.13)	0.0088***(5.08)
Gov	0(-0.08)	-0.0029***(-3.34)	0.0009(1.17)	-0.0017***(-2.99)
Duality	0.0092(0.55)	-0.3060***(-9.13)	-0.0296(-1.34)	-0.2467***(-11.61)
Property	-0.0326(-1.07)	-0.1881***(-3.68)	-0.0752*(-1.93)	-0.1734***(-5.35)
MaSize	0.0003***(6.58)	-0.0001(-0.76)	0.0003***(4.7)	-0.0001(-0.96)
Related	-0.0740***(-4.65)	-0.0395(-1.31)	-0.0923***(-4.43)	-0.0356*(-1.86)
Pay_2	0.1781***(4.93)	-0.0333(-0.51)	0.2155***(4.81)	-0.031(-0.74)
Pay_3	0.1950***(10.9)	-0.0387(-1.15)	0.2636***(10.96)	0.0464**(2.17)
Year	Yes	Yes	Yes	Yes
cons	0.6353**(2.51)	0.0461(0.09)	0.9379***(2.86)	1.1228***(3.46)
Adj. R^2^	0.4531	0.2718	0.4410	0.3169

Note:

*, **, *** indicate significant at 10%, 5%, 1% levels, respectively.

## 7. Summary and recommendations

### 7.1 Summary

In this study, we selected a sample of 147 M&A events of Chinese media companies in the process of transformation and upgrading to examine the impact of the executive compensation system on M&A performance in terms of monetary compensation, equity compensation, and perks, to investigate whether the executive compensation system has an incentive effect. We found that the monetary compensation system has no significant incentive effect on media companies’ transformation and upgrading process. The incentive effects of both the equity compensation and perks show an inverted U-shaped nonlinear relationship. In transforming and upgrading media companies, executives may increase their power and salaries by expanding the company’s size and increasing external investment. Therefore, media companies need to improve and strengthen the remuneration committee system during the transformation and upgrading and dynamically adjust the salaries of executives based on the long-term and short-term results of the transformation and upgrading. When formulating equity compensation, the shareholders of media companies should not only consider the incentive effect of giving executives a certain amount of equity but also be wary of the lack of supervision and other problems caused by the change of power brought about by executives’ shareholding. Perks can be an implicit incentive to complement the explicit executive compensation system. However, media companies need to implement strict rules and regulations on perks to prevent the negative consequences of excessive on-the-job spending.

### 7.2 Recommendations

Under the national strategic plan of deep media integration, Chinese media enterprises have entered the development stage of comprehensive transformation [82]. Media enterprises take measures such as improving internal supervision mechanisms and optimizing internal governance structure to mitigate the negative impact of principal-agent issues and ensure the continuous improvement of enterprise value. In this process, the executive compensation system is constantly improved, and an effective Executive compensation system is implemented in daily operational activities. An effective executive compensation system plays an essential role in stimulating the enthusiasm and responsibility of executives, enabling managers to make long-term efforts to reform media content and organization and enhance the competitive advantage of enterprises.

Media enterprises should establish a monetary compensation system based on performance evaluation, supplemented by risk compensation. According to the statistics on the average salary of executives (Fig 1), the average salary of executives in media companies is higher than that of executives in the A-share listed companies after 2015. It indicates that after the comprehensive transformation of China’s media industry in 2014, media companies began to increase monetary compensation for executives, but there was no significant incentive effect. Therefore, media companies need to improve the performance compensation evaluation system, unify evaluation standards, and achieve dynamic adjustment of executives’ monetary compensation based on enterprise performance. At the same time, considering the characteristics of transformation and upgrading, such as high risk and lagging performance growth, it is also necessary to establish a risk compensation mechanism to enhance executives’ enthusiasm in the implementation process of transformation. Regarding the possible salary limit policies in state-owned media companies, relevant government departments may consider increasing the maximum salary for executives of state-owned media listed companies or delegating the establishment of the monetary compensation system to listed companies to comply with the role of market mechanisms.

Media companies should improve the equity compensation system to prevent the formation of "internal control". In the process of transformation and upgrading, media companies should set the appropriate number of equity grants and strict exercise (unlocking) terms and strengthen the monitoring role of shareholders. By developing an effective monitoring system to prevent "insider control". On this basis, it can enrich the forms of equity compensation, such as current stock incentives, future stock incentives, and option incentives. In transforming and upgrading of media companies, different forms of equity compensation are given to executives based on the characteristics of corporate behavior to ensure the effectiveness of equity compensation incentives.

Media companies should publish detailed information on executive compensation and enhance internal and external supervision of perks. Chinese media companies need a comprehensive disclosure mechanism for executive compensation to reduce information asymmetry. For example, the average executive compensation disclosed by Hubei Broadcasting and Television Company in 2012 was only 5500 yuan, a massive gap from the data disclosed by other media companies. It specifically manifests the incomplete and opaque executive compensation information disclosure system. We can refer to the practice of the United States Securities and Exchange Commission (SEC), which obliges companies to list the total compensation of the top five top executives in their power of attorney and specify the benefits enjoyed by the executives, as well as the stock options salary and bonus. Under a strict information disclosure mechanism, the market and public opinion can effectively conduct external supervision over media company executives. At the same time, media companies should also strengthen the internal management of executives’ perks, improve the financial system and urge executives to regulate their behavior.

Media companies should implement stock option incentives and improve the inside-debt incentive system. In the process of corporate transition and upgrade, executives must be brave to take risks and manage risks well to obtain corresponding compensation for market value. Therefore, media companies could implement stock option incentives for executives in the process of transformation and upgrading, which can promote the risk-taking spirit of executives and rapidly improve short-term performance. At the same time, because of the borderline of risk-taking, i.e., the relationship between market performance and risk-taking will change when the risk-taking exceeds the limit [88] while implementing stock options, the inside-debt incentive schemes, including pension and deferred salary should be implemented to weaken the short-sighted and high-risk preferences of executives that stock option incentives may cause. Although the Chinese government has formulated a creditor’s rights incentive system, including deferred payment of compensation and inside-debt incentive system, in the *Compensation System Reform Plan for executives of State-owned Enterprises* promulgated in 2014, only a few state-owned enterprises currently adopt the system. Therefore, Chinese media companies need to improve the inside-debt incentive system based on existing policies to enhance the manageability of risk-taking in the process of transformation and upgrading.

### 7.3 Limitations and prospects

This study has the following limitations: (1) The relationship between different executive compensation systems was investigated separately, but the interaction between the three executive compensation systems was not examined; (2) This study only took the nature of property rights as a control variable and did not examine the executive compensation system of media companies with different property rights. These limitations of this study do not affect the accuracy of the conclusions and inspire further study.

## Supporting information

S1 Dataset The data set used in this article for discussion and analysis. (ZIP)
